# Behind the Scenes of Parents Nurturing a Child with Autism: A Qualitative Study in Malaysia

**DOI:** 10.3390/ijerph18168532

**Published:** 2021-08-12

**Authors:** Wan Natrah Wan Yaacob, Lili Husniati Yaacob, Rosediani Muhamad, Maryam Mohd Zulkifli

**Affiliations:** Department of Family Medicine, School of Medical Sciences, Universiti Sains Malaysia, Kota Bharu 16150, Kelantan, Malaysia; natrah.64@gmail.com (W.N.W.Y.); maryammz@usm.my (M.M.Z.)

**Keywords:** autism, parents, challenges

## Abstract

Many parents have experienced difficulties in parenting children with autism. We, therefore, consider a more in-depth understanding that is necessary to explore the challenges facing parents and families to provide a better outcome for both. We interviewed 21 parents of 24 children with autism spectrum disorder (ASD) to qualitatively explore the challenges they experienced through a phenomenological framework. Four main aspects emerged as challenges to the parents: inadequate knowledge, psychological distress and stigma, lack of support, and barriers to services. These four themes reflect a lack of balance between the needs of caregivers and the services and resources or support available in the community to meet those needs. Our study contributes to an understanding of how parents perceive challenges, making it easier to take necessary action to meet their needs and ease their burden of stress. A concerted effort is needed to coordinate services across all disciplines to address these challenges.

## 1. Introduction

For most parents, caring for a child is a joyful and challenging experience. However, many studies demonstrate that parents of children with autism spectrum disorder (ASD) are confronted with daunting challenges and an increased risk of psychological distress. ASD is a chronic disorder that requires parents to dedicate a considerable amount of time to care for the affected child. Previous studies have shown that parents found it challenging to deal with the behavioral deficits, restricted social interaction and communication, and strict ritualistic behaviors of children with ASD [1,2]. Individuals with ASD can also have a secondary diagnosis, such as Attention Deficit Hyperactive Disorder or intellectual disability that can complicate parenting and rendering the experience among parents highly variable [3]. Findings from previous studies have also shown that the presence of children with ASD in the family influences different aspects of parental and family life, such as marital relationships, sibling relationships, family socialization patterns, and daily routine [4]. Furthermore, a review of the literature revealed that parents with children who have ASD experienced higher stress levels [2,5] and anxiety and depression [6], felt isolated [7,8], and had a decreased quality of life [9] compared with parents of a normally developing child [10] or one with other disabilities, such as Down Syndrome [11]. These issues were compounded by the child’s maladaptive behaviors, regular outbursts, hyperactivity, aggression, repetitive behaviors, and lack of social interaction [4]. Additionally, because many people did not readily embrace the child’s idiosyncratic behaviors, parents of children with ASD were more susceptible to stigmatization, leading to poor wellbeing [12,13].

Previous studies have also pointed to the inadequate number of research on autism conducted in suburban and rural areas as most parental stress and wellbeing research was conducted among urban parents [14]. This disparity indicates a greater awareness of and support for the autism community in those areas [15]. In contrast, research exploring the experiences of families of children with disabilities living in rural areas are limited [16]. Parents in these areas have different cultural beliefs that have influenced their understanding of ASD as well as their decision-making [8] and accounted for inadequate knowledge and a higher level of stigma among parents and in society [7]. These areas have also suffered from an imbalance in access to reliable information and resources. Most public and private centers for children with ASD are concentrated in urban areas [17]. The lack of ASD-related facilities in rural and suburban areas could lead to delayed assessment, diagnosis, and treatment of children. Other studies have reported service-related obstacles such as long waiting lists for appointments in government-sponsored hospitals and parental dissatisfaction with the diagnosis [18].

**Figure 1:**
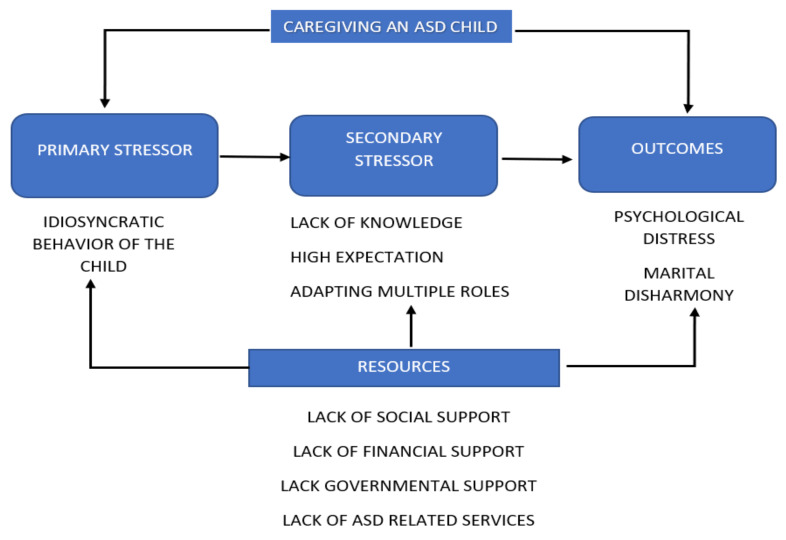
Stressors faced by parents based on the stress process model.

The stress process model (SPM) was suggested as helpful in understanding in-depth the many interacting factors contributing to the experience of parenting a child with ASD [19]. The SPM identifies five main interacting domains that contribute to the parenting outcome. These domains are characterized by five broad theoretical concepts, namely, (a) background and contextual characteristics of parents of children with ASD; (b) objective and subjective primary stressors; (c) parental roles and intrapsychic secondary strains; (d) internal and external mediators; and (e) parents’ wellbeing outcomes [19]. According to this model, combined with the inadequacy of resources to deal with the stressor successfully, a parent’s appraisal of a stressor as a problem will lead to maladaptive outcomes. Consequently, this mismatch between parents’ needs and environmental support may lead to the perception among parents that their situation has surpassed their resources, which could lead to them feeling burdened and overwhelmed [20]. When parents can overcome stressors, parental wellbeing occurs, provided that the potential hurdles have been removed through appropriate support (Figure 1).

This study explores the challenges faced by parents of children with ASD in Kelantan, a relatively low-income and conservative state. To our knowledge, local qualitative studies on families with children with ASD are still scarce. An understanding of their experiences is critical because it provides an overview of daily family realities and how ASD can impact the entire family.

## 2. Materials and Methods

We used a qualitative approach to explore the challenges of parents taking care of their children with ASD. Qualitative research is recognized as an appropriate design for obtaining valuable information when exploring subjective experiences and capturing the complexities of in-depth experiences, such as those of children with disabilities [21]. Aiming at maximum intuitive presentation, we applied the phenomenological methodology to explore and concretely describe the experiences of the parent while maintaining a sensitivity to local context and meaning and remaining as free as possible from unexamined assumptions [18]. Parents who were primary caregivers of children diagnosed with ASD and were able to converse in the local language were included in the study. All subjects gave their informed consent for inclusion before they participated in the study. The study was conducted in accordance with the Declaration of Helsinki, and the protocol was approved by the Ethics Committee of Universiti Sains Malaysia (USM/JEPeM/18050243).

### 2.1. Setting

We conducted the study in Kelantan, located in the north-eastern part of the Malaysian peninsula, with a mixture of ethnicities that included Malay, Chinese, Indian, and minorities such as Siamese. Because of Kelantan’s relative isolation and a mostly suburban and rural lifestyle, Kelantanese culture differs somewhat from the culture of the rest of the peninsula.

### 2.2. Participants

We recruited 21 parents of 24 children confirmed with ASD (aged 18 and below) via our key informants, specialists at psychiatric clinics, and therapists at the rehabilitation centre, HUSM, between February 2019 and July 2019. We screened them for suitability for the study and invited them by letter via these key informants. The contact numbers of parents who agreed to participate were given to the key informants for the first author to contact via telephone. Once the parents had confirmed their participation in the study, we made an appointment with them. We continued with the recruitment of participants until we obtained data saturation [22].

### 2.3. Procedure

After obtaining written consent, the first author held a face-to-face, in-depth interview with each parent. The interviews were held in Kelantanese Malay dialect and recorded using 2 digital voice recorders. Most of the parents preferred to be interviewed at the hospital during the child’s follow-up or during therapy sessions. The average duration of the interview was between 1 to 2 h. During the interview sessions, participant–researcher rapport was established. After introducing themself, the researcher informed the participants that their participation was completely voluntary.

Participants were asked to supply their sociodemographic details. Then, the interview proceeded with open-ended questions exploring work–family problems to build rapport and trust. Subsequently, parents were questioned about the factors making their lives difficult or challenging when raising a child with autism and what kind of support they wanted most. At the end of the interview, the interviewer asked participants to write about their challenges and struggles and how to handle these challenges. She then gave them another 30 min to write about any related issues. We used this complementary approach to improve the reliability of interview data and obtain additional yet relevant insights into the experiences of parents that may have been overlooked during the interview. For each interview, we wrote the field notes based on our interpretation, knowledge, and interaction with the interviewee during the interview sessions, and these notes were important to help us understand the phenomenon better. Each document was transcribed verbatim.

The first author first piloted the interviews among the first 3 parents under the supervision of our senior researcher, who is well trained in qualitative research. We conducted the pilot study to ensure the suitability and comprehensibility of the semi-structured interview guide and to provide the same set of important questions with a detailed account of the parents’ experience. We included data from 2 of the pilot interviews in the analysis because of the rich data obtained. We invited these parents to join the formal study and subjected them to a second interview session and an additional procedure to ensure in-depth discussion and documentation of their account of their life experience.

### 2.4. Data Analysis

We conducted a thematic analysis to identify themes in participants’ responses. We entered the transcripts and the data from the essays into NVivo^®^ to facilitate data analysis. First, the three researchers (WN, LHY, and MMZ) critically re-read the first five transcripts to familiarize themselves with parents’ comprehensive meaning and perspectives. The first author then created a preliminary list of nodes in the NVivo^®^, and we coded the transcripts. We initially analyzed all transcripts individually before considering the identified themes together as a whole to form an overall group analysis and organized them into interconnected hierarchies (that is, themes, subthemes, and categories). To increase trustworthiness and check for coding accuracy, the research supervisors (LHY and MMZ) reviewed the preliminary units of the code of meaning for all interview transcripts. They compared data analysis and themes against a consistency check, resolving any differences through discussion. Additionally, RDM, an expert qualitative researcher, after first reviewing the coding units for 5 transcripts and the themes/subjects for the overall sample, provided general comments and suggestions. We finally reached a consensus within the research team, agreeing on the final codes and the themes, subthemes, and categories. We emailed all the transcripts and final emergent themes back to all parents for validation and confirmation. None of them disagreed with these reports.

## 3. Results

We enrolled 15 mothers and 6 fathers in this study. The parents’ mean age was 38 years (range = 29–54 years). One mother had twins aged 5 years with ASD, while all three of another mother’s children had ASD. The age of the children ranged from 2–14 years. Table 1 depicts the sociodemographic characteristics of the interviewed parents. The findings reflected four main challenges the parents faced while caring for their ASD children. These included inadequate knowledge, psychological distress and stigma, lack of support, and barriers to relevant services.

### 3.1. Inadequate Knowledge

Inadequate knowledge is a critical concern in treating a child with autism, and it occurs in parents, healthcare providers, and in the community. Parents expressed concern about the lack of understanding of the disorder causing delays in seeking help for their child, the absence of services providing appropriate management, and the community response to a child with autism and their parents.

The challenges of autism were something new for these parents. Nearly half had never learned about autism until their child was diagnosed. None could give autism its exact meaning. Even those who had heard of autism were not interested in understanding it in any depth, although their child had some features related to autism.

Now I know a bit about it. I know that kids with speech delay could be autistic. However, I was not sure about this, and I did not research as to why my kid is like this; is he autistic or what? [P7]

Because certain features are only apparent when children reach a certain age and are sometimes subtle, a lack of knowledge and awareness among these parents about ASD often contributes to delays in identifying their child’s symptoms. Some parents noticed that the child was different only when informed by others (for instance, neighbors, friends, or doctors).

Four years ago, I had my neighbor looking after my son. I told her that my son would not go anywhere; he would only sit there. She said he could be autistic. I asked her, “What is that?” I did not know what autism was. [P14]

Parents also delayed seeking related information because of their social norms and cultural background. To some parents, speech delay was accepted as their family norm because some members of their family, particularly boys, had speech delays at that age and had later become normal. Parents did not bother to investigate autism further.

Before this, we tended to ignore it, and it is common; you know how the elderly are … even my mom said, “That is not unusual. Even your siblings, some of them were like that too.” So we think this is normal. [P9]

Some parents claimed that living in remote areas was another disadvantage that impacted their ability to access autism information. They were not sure where to go and who to ask about the child’s condition. They needed to ask around, discuss the matter with close friends and neighbors, search for information on the Internet, or read the information on Facebook. They said the attempt to obtain information about their child’s condition was a time-consuming, frustrating process.

You know how it is here (suburban area), the awareness of autism is quite low. For example, in my child’s case, I only know now. Only here (from this hospital), I have learned that there are many autistic kids. Most of those who are affected do not know where to seek help or go for therapy. [P6]

Parents also reported that awareness and knowledge of autism among the community was poor. Their children were often labeled as anti-social. Parents found it hard to change public misconceptions, especially when dealing with people in rural areas.

When my son cannot speak, the people around him immediately think that he is mute and deaf. So, we have to explain to them that our son is not deaf or mute; he prefers to be immersed in his world. [P15]

Additionally, some parents realized that health care providers’ knowledge of autism was also inadequate. P5 reported that some health care providers were not equipped to deal with and treat children with autism: “Even the doctors did not know what to do. They did not know much about autism. The only thing they know is sending our child to therapists.”

### 3.2. Psychological Distress and Stigma

Caring for a child with ASD is an emotionally exhausting experience. Parents shared a wide range of emotional challenges they had faced from pre-diagnosis to the present. At the early stage of detecting features related to autism, the parents were confused and had difficulty understanding their child’s behavior. Parents struggled with fear and apprehension about their children’s differences and tried to resolve their children’s problems on their own. P7 explained her perplexity and misery as follows: “I am sad because he is not like other kids. He is trapped in his world. I cannot understand him. I am sad with this situation”.

At diagnosis, parents expressed their grief as denial and disappointment. Many parents were wary of acknowledging their children as having ASD. They wanted more time to accept the news and comforted themselves by denying reality and avoiding therapy for their child. These parents also reported disappointment after the doctor’s confirmation. Discussing the future and calling their child “OKU,” or disabled, was a heart-breaking experience for many parents. They felt a deep sense of loss as they had to give up their last hope of being “normal” and accept living with a disorder. The lack of awareness and inadequate support to treat disabilities aggravated their misery and depression as reported by P17: “The doctor asked me if I wanted to have an OKU (person with a disability) card (for my child). I felt so down at that time. I cried so hard after I came back from the clinic”.

Consequently, after the diagnosis, the parents experienced self-reflection and felt guilty that they had caused their child to have ASD. P1 accused herself of expecting her son at an older age, saying, “I did blame myself initially—getting pregnant at 40, with a 10-year gap between him and his sister.” In contrast, P18 thought it was God’s punishment for her past mistakes.

In addition to the emotional upheaval they faced early in the diagnosis, both parents expressed their day-to-day emotional distress in dealing with their child’s idiosyncratic behaviors. They described the child’s hyperactivity, inattentiveness, abnormal ritualistic habits, tantrums, self-harming behaviors, and lack of communication skills as the major source of stress and anger among them. P15 said, “Of course, we accept them, but when we are alone with them and they throw tantrums, yes, we can feel down too.” Sometimes they had to deal with abnormal sleeping patterns and weird obsessions, which can be mentally exhausting and physically tiresome. One mother disclosed, “My son spends most of his time with his babysitter; he does not sleep much at night. Once, he did not sleep for three days straight.” [P6]

Twelve parents spoke about the frequent worries caused by the unforeseen and potentially harmful and dangerous actions of their children who required extra care and attention.

It is tiring because I need to look after the house on top of looking after him. I cannot leave him alone. I am scared if I leave him unattended, he will climb over the metal bar and go upstairs and get hurt. So, he needs constant attention. [P12]

Apart from the child’s challenging behaviors, parents complained about the other responsibilities they had to perform. Parents spoke of their responsibility to attend the child’s counseling appointments and follow-up to ensure the child’s progress. The child with ASD needs constant teaching and learning, and parents were often required to take responsibility and act as instructors and therapists at home. They were aware that they could not concentrate on being just a mother/father and leave the autism and activities of their child to health care professionals and their teachers. P4 shared that “Having a child with special needs is very exhausting. We must deal with so many things. Their therapy, their growth, their improvement.”

The parents also mentioned the burden of the many duties they had to handle at home or at work. Most of them had work obligations that were often nearly as overwhelming and stressful, particularly without a supportive network. The emotional burden also increased when other children were also hard to handle.

Besides their psychological distress, stigma caused emotional challenges. “Embarrassed” was the word most frequently used by parents while raising children with ASD. Most parents considered public tantrums to be the most challenging aspect of their children’s behavior. Parents reflected on how the perceived opinions of others were more frustrating than the behavior itself. Parents generally thought that others tended to judge, not acknowledge, their parenting abilities.

When I bring him to the store, people tell me to make him behave. Before I take him anywhere, I will tell him, “Listen, we are going to a public place, so you have to behave yourself.” When I walk to the car, I remind him again, but at the end of the day, he still does what he wants. [P9]

Unforeseeable tantrums attracted people’s attention and caused them to react negatively. It gave the impression to others that the child was misbehaving and portrayed them as bad parents as relayed to us by P17, “People would frown, thinking I was a bad parent, ignoring my kid screaming like that.” Another issue that parents found embarrassing was the OKU card or disability card. Although they recognized that the card might be beneficial to their child, they were concerned that it would become a source of stigma. This was mentioned by P10: “Thinking about getting an OKU card made us feel more uncomfortable that people might think my child is abnormal, so we rejected the idea.”

Half of the children in our study had experienced isolation and rejection, especially during social events. A few parents felt humiliated when their children were not listed for or were excluded from their school activities. P16 told us, “The teachers once mentioned organizing a field trip but then they said that my child did not need to attend.”

Some of the parents even received complaints and nasty comments from others, which were much more difficult to ignore and often made the parents sad, causing them to react to mollify the situation.

My nephew said that my son was crazy; I mean, I know he is just a kid, and of course, kids do notice these things, especially when he got so excited he would make his butt and his face look weird. [P1]

### 3.3. Lack of Support

The parents expressed the need for support from others, especially from the family, community, or government, to help them cope with parenting responsibilities. Eight parents identified at least one family member who did not attempt to understand autism and failed to offer support. P6 sadly said, “I am tired. With an unhelpful husband, who tends just to ignore things. I have to do everything on my own.”

A few parents felt disconnected from their spouses, especially when they had conflicting views about a child’s care and upbringing.

I told him that I wanted to bring our son to a psychiatric clinic because of his delayed speech. But he asked why? He claimed his son was normal. Because his children with his other wife are all normal. [P18]

A few others shared the consequences of lacking the support of a husband. This had exacerbated the conflict and disharmony between them and even led them to apply for divorce.

I asked for a divorce because he never comes home, and even if he does, he only comes home at night for two hours. That is why I said I am better off on my own. So, I take it. Let us just go our separate ways because when my son is sick, it has always been me alone. [P18]

Having support from other children is also quite challenging. Some children, rather than understanding what had happened to their sibling, were jealous of the child with ASD. Parents also experienced challenges in creating a stable and loving family environment.

I told my elder daughter that she should have pity on her young brother. She said that Mama is always sorry for him, but Mama doesn’t pity her. I said, “I treat all of you the same. But your brother is the different one. If he were the same as you, I would treat you two equally.” I’ve been trying to make her understand. She’s envious. [P13]

Parents also felt alienated from their extended family members, who seemed to lack an understanding of their children’s disorder. They had not even provided emotional support and physical assistance when needed. Some family members had shown inappropriate reactions when dealing with these children. P3 illustrated the event as follows: “When I was packing up, I heard everyone (the relatives) scolding my son (with nasty words) like ‘I will whip you.’ Some even shook him.”

While caring for children with ASD, many parents (*n* = 15) experienced financial hardship because of expensive professional services (such as nursery and preschool), childminders, special education, long-distance service providers, and other necessities (for instance, milk and diapers). P4 shared her challenges; “It is a struggle because we’re trying to pay off children’s fees that are so expensive, on top of our monthly expenses. It’s a struggle”.

Many families were forced to make considerable lifestyle adjustments and job changes that further caused financial instability among these parents. They expressed their need for more government support to reduce their financial burden while caring for children with ASD as relayed to us by P6: “The government needs to expand their services to increase funds for autism because not all parents are well off.”

Additionally, almost all parents were concerned about the child’s long-term future. While parents were struggling to equip their children with the skills needed to make them independent and to survive in the community, they were also concerned about who would take care of their child when they died. P9 tearfully said, “I hope that he would be able to be independent, be able to take care of himself, able to survive on his own.” Unfortunately, many parents felt that there was inadequate support from the government that could guarantee the future of these children, especially as regards financial support, education, and future careers. They were unsure where to channel their children after secondary school. They hoped for a brighter career pathway for children with ASD.

My concern now is his life direction. He is already 14 years old. What will happen after he finishes school? I am constantly thinking about his future. If possible, I don’t want him to stay at home, but to go out there and do something for himself. To freely choose his passion for cooking. That is what I am aiming for at this moment. [P15]

### 3.4. Barriers to Relevant Services

Children with disabilities rely heavily on important professional services to help them improve their functioning. However, many parents have experienced challenges in finding professional therapy services. Few effective therapies were available in rural areas. As P20 noted, “One thing we can see in Kelantan is that there is no specific place for autism parents and autistic kids. In Kuala Lumpur, there are many specific schools such as Permata Kurnia.” Parents had to travel a long way to obtain treatment. Otherwise, the children had to be sent to a private center, which was very expensive.

Other parents complained about common government-sponsored hospital issues such as long queues for the first appointment, long waiting hours, lack of intervention slots, lack of parking spaces for disabled people, and complex follow-up procedures. P7 commented:

We had to wait for a long time before getting to see the specialist, until my son’s diaper was all wet and full. It is also tough to find parking here, and if we park at the wrong place, we will be fined RM30.

Some of the parents shared their dissatisfaction regarding ineffective communication between health care providers and their inability to recognize autistic features despite parental concern.

Apart from related therapeutic services, half of the parents spoke about their continued struggle to fight for their child’s education and find an appropriate school. One of the parents pointed out her frustration following her son’s school rejection and difficulties in finding a suitable school.

We sent him to a government kindergarten. The teacher could not handle him. The teacher said he could not sit still. The teacher said that he was not disturbing, but he wandered around. Then the teacher said that he did not fit in at that school. [P16]

There was also limited awareness and knowledge among the teachers, who were unaware of autism and did not know how to deal with children who had autism. More worryingly, parents reported mistreatment of autistic children in those schools. As P9 angrily said, “For instance, a lunch box and trousers stained with feces should not be put together. For me, this is not right. My son was locked in another room. His ear was twisted and pulled.”

Parents with young children were also concerned about enrolling their children in mainstream schools, fearing that the crowded and unfavorable conditions would put their child at risk of neglect and isolation from others. They were also concerned about the lack of an appropriate school curriculum and autism assessment tools to assess the children’s learning process accurately.

The teacher doesn’t know how to evaluate, and she doesn’t know whether my child can read or not. She seems to have taught my child the same thing as kids with Down Syndrome. [P20]

## 4. Discussion

Our study demonstrated the interrelations of parental stress indicators present in the SPM of parental caregiving in response to ASD. The findings revealed four significant challenges: inadequate knowledge, psychological distress and stigma, lack of support, and barriers to relevant services. Before diagnosis, most of the parents in this study had scant knowledge of autism, consistent with the findings of Dolah et al. [23]. The acquisition of accurate knowledge of autism was affected by many factors, including cultural beliefs and practices. Cultural beliefs affected how parents perceived the symptoms of ASD, thereby determining their subsequent responses [5]. Many of these parents perceived the child’s symptoms as acceptable and falling within the bounds of normalcy, thereby delaying the search for relevant information about autism [24]. Additionally, mental health awareness on ASD is still growing in Asia and is in the early stages of receiving the necessary support [15]. Similarly, the fact that parents came from Kelantan, a less developed state than Kuala Lumpur (Malaysia’s capital) also impacted their awareness of autism [25]. This condition explains the lack of knowledge among parents, the community, and even professionals such as teachers and health care providers in this study.

Our study highlighted the psychological distress suffered by parents. In the beginning, the lack of parental skills and the inability to comprehend the child’s development led to confusion and apprehension, particularly before confirmation of the diagnosis. Parents experienced fear and anxiety about the eventual diagnosis of ASD in their children. During diagnosis disclosure, we discovered mixed emotional reactions among parents, including denial, grief, guilt, and disappointment when their expectation for the child to be normal was diminished. Subsequently, some parents were overwhelmed by guilt for being the cause of their child having autism. They felt at a loss about what to do next [18,26,27,28].

Past research has shown that a child’s behaviors and the severity of ASD contribute to parental stress and wellbeing [29]. Our results confirmed that psychological distress was directly related to caring tasks, exacerbated by a high level of unforeseen behavioral challenges, compounded by the child’s lack of communication and minimal engagement with the child. The child’s behavior was frequently erratic, often leading to tantrums and aggression. Parents also addressed hyperactivity and sleep problems, indicating the importance of potential attempts to tackle these behavioral impacts on parental wellbeing [29]. The lifelong condition and the dependence of the child on the care of others also increased the stress on the parents. Role strain in this study was caused by the multiple parental roles to which they had to adapt in helping the child with ASD achieve normal growth and development [27].

Parents in the study also experienced stigma in their daily lives [12] and felt that stigma is typified by feelings of shame or the fear of rejection because of the child. Enacted stigma refers to instances of overt rejection or discrimination experienced by stigmatized individuals [13]. Often the misbehavior of the child contributes to that feeling among parents. This study uniquely encountered the term “OKU” used repeatedly by the parents. “OKU” is the term for groups of disabled people in this country. Registering their child as an OKU and having the OKU card is a source of stigma to the parents. Therefore, some parents were reluctant and had misgivings about the OKU card despite the many benefits it offered in terms of accessibility to facilities. It is difficult for other people to recognize children with ASD as having disabilities because of their normal appearance. Consequently, parents were frequently criticized as bad parents by others when their children misbehaved because they were unable to control them [27]. Previous studies referred to this experience as social isolation, community intolerance, and difficulties in negotiating public encounters [7]. Parents in our study were commonly rejected and isolated by the community members. This included exclusion from school activities and derogatory remarks by community members, resulting in parents limiting their outside activities, which most parents found distressing.

Lack of support was another important challenge facing parents. Support is a fundamental aspect of the wellbeing of caregivers. While a few families had a support system, most parents experienced a non-supportive response from family members, who failed to understand their child’s condition and ultimately failed to help whenever needed. Consequently, parents experienced health impacts and disharmony in relationships where other children felt neglected [30], and the marriage was compromised [11]. Our study confirms the findings of recent studies indicating that parents of disabled children frequently experience a profound lack of family support to function optimally as a parent [20].

Parents are further challenged by a lack of financial support and support for their children’s future. Rogge and Janssen attributed the financial burden to the daily cost of basic needs (such as milk and diapers) and the expense of therapies and special schools [31]. Clinical therapy for ASD involved multidisciplinary professional visits that led to time and financial constraints for these families [32], exacerbated by the difficulty of finding an understanding childminder during working hours, which in turn caused parents to resign from their current employment in search of a more flexible workplace [33]. Collectively, this affected their ability to work, thus increasing the financial burden on the entire family [34]. Almost all parents in this study had encountered problems in securing their child’s future. Their concerns about both the future care of the child after their death and the child’s future education as well as career paths led to stress. Parents’ anxieties and concerns about the child’s future reflected the lack of resources and support services available in the region to manage ASD [17]. This finding suggested that parents in the region were faced with a lack of essential resources and support services.

Parents also highlighted their difficulties while trying to access services for their children with ASD [28]. On the journey to “treat” the child, parents experienced delays in confirming the diagnosis, due in part to the failure by health care workers to recognize subtle features related to ASD, despite parental concern regarding their child’s differences [26]. Ineffective communication between healthcare providers and parents during the consultation led to parental dissatisfaction and affected their compliance with scheduled treatment [35]. Other challenges included long waits for a first appointment, lack of treatment slots, lack of parking lots for the disabled, and complicated policies and bureaucracies, findings similar to those of Chu et al. [32].

We recognize the limitations of our study. Despite efforts to involve both fathers and mothers of different ethnic backgrounds, the number of participants representing each ethnic group was inadequate. However, it reflected the true ethnic proportions in Kelantan, where 98% are Malays, and the majority of main carers for children were their mothers. Another area that should have been highlighted is the socioeconomic background of the carers that could be an important factor in their experience raising children with autism. Information regarding the children’s schooling and detail on their treatment should have also been examined to provide us with a better understanding of the whole experience. We recommend that these areas need to be included in future research on the topic.

## 5. Conclusions

The SPM facilitates an understanding of the challenges facing parents in caring for ASD children while trying to maintain their wellbeing. Our results suggest that parents of children with ASD experience daily challenges within four themes: inadequate knowledge, psychological distress and stigma, lack of support, and barriers to relevant services. Better recognition of the various associated stresses and stressors for families will create services better designed to support those already dealing with the personal and social challenges of raising an ASD child. We recommend that improvement in the education of medical professions, teachers, family members, and community regarding ASD is severely needed to improve the overall understanding and care of children with ASD. In addition, we recommend the use of telemedicine to tackle the need of those from rural areas. Furthermore, there also needs to be a coordination of care between all the different parties involved in the management of children with ASD. Finally, a better support structure in terms of financial and psychological aspects are also required to ensure that parents have the necessary assistance they need.

## Figures and Tables

**Table 1:** Characteristics of study participants.

ID	Age	Ethnicity	Occupation	Marital Status	Age of Child (Year)	Age of Diagnosis (Year)
P1	41	Malay	Housewife	Married	3	1.5
P2	43	Malay	Driver	Divorced	4	2.5
P3	37	Malay	Housewife	Married	3 y	3 y
P4	38	Malay	Housewife	Married	7/5/2	4/2/2
P5	38	Malay	Barber	Married	6	2.5
P6	41	Siamese	Freelance accountant	Married	5	4
P7	32	Malay	Nurse assistant	Married	4	2
P8	33	Malay	Counselor	Married	7	2
P9	39	Malay	Businessman	Married	9	5
P10	36	Malay	Housewife	Married	7	3
P11	35	Malay	Housewife	Married	5/5	5
P12	38	Chinese	Businesswoman	Married	9	3
P13	32	Malay	Housewife	Married	7	4
P14	40	Malay	Gym trainer	Married	6	1.5
P15	54	Malay	Housewife	Married	14	3
P16	36	Malay	Assistant auditor	Married	7	5
P17	32	Malay	Research assistant	Married	5	2.5
P18	29	Malay	Housewife	Married	7	4
P19	36	Malay	Fruit seller	Married	8	2
P20	51	Malay	Physiotherapist	Married	14	2
P21	31	Malay	Housewife	Married	5	2

## Data Availability

Data are contained within the article.
